# Clinical features of intestinal tuberculosis: an analysis of 46 cases, including six initially misdiagnosed as Crohn's disease

**DOI:** 10.3389/fmed.2026.1921992

**Published:** 2026-09-10

**Authors:** Lin Li, Jing Wang, Xiaoping Niu

**Affiliations:** Department of Gastroenterology, The First Affiliated Hospital of Wannan Medical University, Wuhu, China

**Keywords:** clinical analysis, Crohn's disease, endoscopy, intestinal tuberculosis, misdiagnosis

## Abstract

**Objective:**

The purpose of this study was to summarize the clinical, laboratory, endoscopic, imaging, and histopathological features of intestinal tuberculosis (ITB), with a focus on the clinical characteristics of ITB cases initially misdiagnosed as Crohn's disease (CD), to improve clinicians' understanding and diagnosis of ITB.

**Methods:**

The clinical data of patients diagnosed with ITB at the First Affiliated Hospital of Wannan Medical University between 1 January 2012 and 31 December 2025 were retrospectively analyzed. Among them, six patients were initially misdiagnosed as having CD and were analyzed as a separate subgroup. All data were analyzed using descriptive statistical methods.

**Results:**

In total, 46 patients with ITB were enrolled in the study. The most common clinical manifestation was abdominal pain (32 cases, 69.56%), followed by abdominal distention (18 cases, 39.13%). The positive rates of the T-Spot assay for tuberculosis (T-SPOT.TB test), purified protein-derived (PPD) test, and chest imaging were 81.25% (26/32), 40.91% (9/22), and 39.13% (18/46), respectively. In the subgroup of six patients with ITB initially misdiagnosed as CD, all six underwent PPD and T-SPOT.TB testing, with positive rates of 50% and 100%, respectively, indicating 50% agreement between the two diagnostic methods. Because ITB could not be ruled out, all six patients received diagnostic antituberculosis therapy and showed a favorable response.

**Conclusion:**

The clinical manifestations of ITB lack specificity, and its endoscopic and imaging manifestations are diverse, making ITB easily confused with CD. In patients with a high clinical suspicion of ITB, diagnostic antituberculosis treatment can serve as an important auxiliary diagnostic method. Improving the recognition of atypical ITB cases may help reduce misdiagnosis and improve patient prognosis.

## Introduction

Intestinal tuberculosis (ITB) is a specific infection caused by *Mycobacterium tuberculosis* (MTB) invasion of the intestinal wall and can be classified as primary or secondary. Clinically, ITB is mostly secondary to tuberculosis (TB), and gastrointestinal TB accounts for approximately 0.15% of gastrointestinal infections (1). Statistics show that 5.9% of patients with TB in the United Kingdom have ITB (2). In recent years, the incidence of ITB has increased along with the increasing incidence of TB worldwide (3). China is one of 22 countries identified as having a high burden of TB (4). Because the clinical manifestations and laboratory findings of ITB lack specificity, misdiagnosis is common. ITB shares many clinical, endoscopic, and pathological features with other gastrointestinal diseases, such as Crohn's disease (CD), intestinal lymphoma, and intestinal Behcet's disease. In particular, CD is a chronic granulomatous intestinal disease that can affect the terminal ileum and colon, and its clinical, endoscopic, imaging, and histopathological manifestations overlap extensively with those of ITB. For instance, ITB can present with longitudinal ulcers, cobblestone-like changes, and other “CD-like” endoscopic features, while CD can also show atypical manifestations such as circular ulcers (5). This overlap leads to a misdiagnosis rate of up to 20%–50% for ITB (6, 7). More importantly, patients with ITB who are misdiagnosed as having CD and receive immunosuppressive treatments such as glucocorticoids or anti-tumor necrosis factor therapy may develop disseminated TB and experience rapid deterioration of their condition, which can be life-threatening (8, 9). To address this issue, researchers have attempted to establish various diagnostic models (5). However, the application of these models in clinical practice remains limited, especially in resource-limited regions. The diagnosis of ITB still relies heavily on the comprehensive judgment and experience of clinicians.

In recent years, studies have found that 40% of patients with ITB develop complications, including intestinal obstruction, intestinal perforation, intestinal fistula, and malabsorption syndrome, and require surgical treatment (10). ITB is a major public health problem in developing countries, causing high morbidity and mortality rates (11–13).

Against this background, the differential diagnosis between ITB and CD remains a key challenge in clinical practice. On the one hand, the endoscopic and histopathological manifestations of ITB are diverse, and some cases may present with atypical changes such as longitudinal ulcers, noncaseating granulomas, or even pebble-like nodules, which substantially overlap with those of CD. On the other hand, because of limitations in sampling methods and detection techniques, the rate of obtaining microbiological evidence in clinical practice is suboptimal, making it difficult to establish a clear etiological diagnosis in some patients with ITB at initial diagnosis. Therefore, a thorough understanding of the clinical manifestations of ITB, particularly the characteristics of atypical cases that can easily be confused with CD, has clear clinical significance. In this retrospective descriptive case series, we collected clinical data from 46 patients with ITB admitted to our hospital between 1 January 2012 and 31 December 2025. We described and analyzed their clinical, imaging, endoscopic, and histopathological features, with a focus on cases initially misdiagnosed as CD, to provide clinicians with information that may improve the recognition of ITB and reduce misdiagnosis.

## Methods

### Study design and participants

This retrospective descriptive case series consecutively included patients diagnosed with ITB at the First Affiliated Hospital of Wannan Medical University between 1 January 2012 and 31 December 2025. All clinical data were obtained from the hospital's electronic medical record system.

The inclusion criteria were a final diagnosis of ITB; complete clinical records, including demographic data, clinical manifestations, laboratory test results, and endoscopic and imaging examinations; and receipt of standard antituberculosis treatment and completion of at least 6 months of follow-up.

The exclusion criteria were an unclear final diagnosis or loss to follow-up and >30% missing clinical data.

### Diagnostic criteria

The diagnostic criteria for ITB were based on relevant domestic and international guidelines and literature (14, 15).

A confirmed diagnosis required any of the following: histopathological evidence of caseating granulomas, microbiological evidence based on positive acid-fast staining or culture, or molecular evidence based on a positive polymerase chain reaction (PCR) or Xpert MTB test of affected tissue.

For a clinical diagnosis in the absence of the above confirmatory evidence, patients were required to have both typical endoscopic/imaging findings and a favorable response to antituberculosis treatment (follow-up ≥6 months).

### Data collection

#### General information and clinical manifestations

Data collected included patient age, sex, history of TB, history of contact with individuals with TB, and main clinical symptoms (e.g., abdominal pain, diarrhea, bloody stool, fever, night sweats, weight loss, and abdominal mass).

#### Laboratory tests

Peripheral blood test results at the time of initial diagnosis (before antituberculosis treatment) were collected, including white blood cell count, hemoglobin, platelet count, albumin, C-reactive protein (CRP), and erythrocyte sedimentation rate (ESR). Increased values were determined according to the normal reference ranges provided by the hospital laboratory. Results of acid-fast staining of sputum smears and intestinal contents, tuberculin skin testing (purified protein derivative [PPD] test), and the T-Spot assay for tuberculosis (T-SPOT.TB) test were also recorded when available.

#### Endoscopic assessment

For patients who underwent colonoscopy, the following endoscopic features were recorded: lesion location (ileocecal region and terminal ileum, terminal ileum, ileocecal region, ascending colon, transverse colon, sigmoid colon, or jejunum), lesion morphology (proliferative, ulcerative, or mixed type) (16, 17), ileocecal valve changes (thickening, deformation, fixation, or incomplete opening), and other findings (intestinal stenosis, fistula formation, and inflammatory polyps).

Typical endoscopic manifestations included circular ulcers, involvement of the ileocecal region, inflammatory polyps, intestinal stenosis, and deformation of the ileocecal valve.

The proliferative type was characterized mainly by granulation tissue hyperplasia and fibrosis. Endoscopic findings included intestinal stenosis, a stiff intestinal wall, pseudopolyp formation, and protruding tumor-like masses. On imaging or endoscopic examination, this type may be confused with intestinal tumors or CD, which is an important cause of misdiagnosis.

The ulcerative type was characterized mainly by mucosal ulceration and necrosis. Endoscopic findings included multiple superficial, circular ulcers perpendicular to the long axis of the intestinal lumen. Systemic symptoms of toxicity were more severe, requiring more intensive infection control and supportive treatment.

#### Imaging assessment

For patients who underwent imaging examinations, the following features were recorded: intestinal wall thickening (defined as thickness >3 mm), ileocecal constriction or deformation, mesenteric lymph node enlargement (defined as a short-axis diameter of >10 mm) and the presence of central necrosis, intestinal stenosis and its morphology (symmetrical/centrifugal), fistula or sinus tract formation, and an abdominal mass.

Typical imaging manifestations included ileocecal constriction, asymmetric thickening of the intestinal wall, enlarged mesenteric lymph nodes, and intestinal stenosis accompanied by dilation of the proximal intestinal segment.

#### Histopathological assessment

All endoscopic biopsy and surgical resection specimens were fixed in 10% neutral formalin, routinely embedded in paraffin, sectioned, and stained with hematoxylin–eosin and acid-fast stains. Two pathologists independently reviewed the slides. The following histological features were recorded: the presence or absence and type of granulomas (caseating vs. noncaseating), granuloma distribution (confluent vs. scattered and isolated), degree of inflammatory infiltration of the submucosa, and presence or absence of acid-fast staining.

Histological diagnostic features of ITB included caseating granulomas, disproportionate submucosal inflammation, band-like infiltration of epithelioid histiocytes at the ulcer margins, and positive acid-fast staining or positive tissue culture/molecular testing (PCR/Xpert).

Histological diagnostic features of CD included noncaseating granulomas, fissuring ulcers, transmural inflammation, nerve fiber proliferation, and distortion of glandular architecture (e.g., crypt branching and Paneth cell metaplasia) (18, 19).

### Statistical analysis

Data were analyzed using IBM SPSS Statistics version 26.0 (IBM Corp., Armonk, NY, USA). Because this was a descriptive analysis, categorical data were expressed as number (*n*) and percentage (%). The normality of continuous data was assessed before analysis. Normally distributed data were expressed as the mean ± standard deviation, whereas nonnormally distributed data were expressed as the median (range).

## Results

### Patient characteristics

A total of 46 patients were enrolled, including 28 male (60.87%) and 18 female (39.13%) patients. The mean age was 47.21 ± 18.47 years for male patients and 48.33 ± 15.39 years for female patients; the male-to-female ratio was 1.6:1. One patient (2.17%) was aged 10–20 years, 27 (58.70%) were aged 20–50 years, and 18 (39.13%) were aged >50 years. The duration of disease ranged from 48 h to 10 years.

### Clinical manifestations

As shown in Table 1, abdominal pain (32/46, 69.56%) was the most common presenting symptom of ITB, followed by abdominal distention (18/46, 39.13%) and diarrhea (10/46, 21.74%). Among patients presenting with abdominal pain, seven had an acute abdomen, including five with intestinal obstruction and two with intestinal perforation. Twenty patients had coexisting extraintestinal TB, including pulmonary TB in 16 (80%), abdominal wall TB in three (15%), and liver node TB in one (5%).

**Table 1:** General data and clinical manifestation of the 46 patients with ITB.

Primary predicted symptoms	Number (*n*)	Percentage (%)
Abdominal pain	32	69.56
Abdominal distention	18	39.13
Nausea and vomiting	4	8.70
Diarrhea	10	21.74
Fever	2	4.35
Fatigue	1	2.17
Emaciation	3	6.52
Bloody stool	3	6.52

Laboratory findings	Number (*n*/*N*)	Percentage (%)
Elevated Leukocyte	7/46	15.22
Amenia	36/46	78.26
Elevated platelets	18/46	39.13
Hypoalbuminemia	34/46	73.91
Fecal occult blood	4/46	8.70
Elevated CRP	13/20	66.70
Elevated ESR	13/20	66.70
Acid-fast bacilli positive in sputum	2/10	20.00
PPD positive	9/22	40.91
T-SPOT.TB test positive	26/32	81.25

Gastrointestinal endoscopy or surgery	Number (*n*)	Percentage (%)
Gastrointestinal endoscopy	27	58.70
Received surgery	24	52.17
Location		
Ileocecal part and terminal ileum	2	4.35
Terminal ileum	6	13.04
Ileocecal part	24	52.17
Ascending colon	5	10.87
Transverse colon	2	4.35
Sigmoid colon	1	2.17
Jejunum	4	8.70
Pathological findings		
Granulomatous inflammation	42	91.30
Caseous necrosis	18	39.13
Chronic inflammation with granulation tissue	1	2.17
Chronic inflammation with massive eosinophilic leukocyte infiltration	1	2.17
Colon malignancy	2	4.35

ITB, intestinal tuberculosis; CRP, C-reactive protein; ESR, erythrocyte sedimentation rate; PPD, purified protein-derived tuberculin, T-SPOT.TB test, tuberculosis infection based on the number of spot-forming T cells.

### Laboratory and imaging examinations

As shown in Table 1, the leukocyte count was elevated in seven patients (15.22%), with a mean of 7.59 ± 3.71 × 10^9^/L. Anemia was present in 36 patients (78.26%), including moderate-to-severe anemia in eight, and the mean hemoglobin level was 106.41 ± 18.50 g/L. Elevated platelet counts were present in 18 patients (39.13%), with a mean of 275.78 ± 114.33 × 10^9^/L. Hypoalbuminemia was present in 34 patients (73.91%), with a mean albumin level of 31.94 ± 6.64 g/L. Fecal occult blood testing was positive in four patients (8.70%). CRP was elevated in 13 of 20 patients (66.7%), with a median CRP level of 28.5 mg/L (range, 8.1–73.6 mg/L). ESR was elevated in 13 of 20 patients (66.70%), with a median ESR of 40.0 mm/h (range, 18–65.8 mm/h); six patients had a markedly elevated ESR (>50 mm/h). Ascites was detected in one patient and was exudative. Sputum was tested for acid-fast bacilli in 10 patients, with positive results in two. The positive rates for the T-SPOT.TB and PPD tests were 81.25% (26/32) and 40.91% (9/22), respectively.

All 46 patients underwent chest imaging, including chest x-ray in 12 and computed tomography (CT) in 34; TB was detected in 18 patients (39.13%). Abdominal imaging was performed in 36 patients, including standing plain radiography in four, abdominal CT in 22, and total gastrointestinal CT angiography in 10. The findings indicated intestinal tumors in five patients, intestinal obstruction in 11, ileocecal thickening in 16, and intestinal TB in four.

### Gastrointestinal endoscopic and pathological findings

Endoscopic and postoperative pathological findings are shown in Table 1. Twenty-seven patients underwent endoscopy. The main lesion types were hypertrophic (59.26%) and ulcerative (48.15%). Endoscopic biopsy showed granulomatous inflammation in 42 cases, caseous necrosis in 18, chronic inflammation with granulation tissue in one, and chronic inflammation with extensive eosinophilic infiltration in one. Among these, five were diagnosed as ITB on endoscopy, two were suspected of ITB, and three were suspected of either ITB or CD.

A total of 24 patients underwent surgery. Overall, lesions involved the ileocecal region and terminal ileum, terminal ileum, ileocecal region, ascending colon, transverse colon, sigmoid colon, and jejunum in 2, 6, 24, 5, 2, 1, and 4 patients, respectively (Table 1).

### Treatment methods and outcomes

ITB was histopathologically confirmed in 26 patients (56.52%), and 8 (17.39%) were diagnosed based on clinical manifestations consistent with ITB and evidence of active extraintestinal TB. Twelve patients received diagnostic antituberculosis therapy with a four-drug regimen consisting of isoniazid, rifampicin, pyrazinamide, and ethambutol. After 6 weeks of treatment, symptoms such as fever, abdominal pain, and diarrhea were significantly relieved. After 6 months of antituberculosis treatment, endoscopy showed significant improvement in intestinal granulomatous hyperplasia and annular ulcers.

Surgical treatment was performed in two patients because of intestinal perforation, one because of intussusception, three because of suspected colon tumors with intestinal obstruction, and 18 because of ileocecal and ascending colon TB with intestinal obstruction. Surgically resected specimens were examined histopathologically, and all were diagnosed as ITB; caseous necrosis was found in 18 patients, including two with concomitant colon malignancy (Table 1). Four-drug antituberculosis therapy was continued for 6 months after surgery.

Two patients died of multiple organ failure after surgery for acute intestinal perforation. The remaining patients received antituberculosis treatment for >6 months and were followed for 6 months to 5 years. No recurrence was reported.

### Subgroup analysis of 6 patients with ITB initially misdiagnosed as CD

As shown in Table 2, the subgroup comprised six patients aged 20–56 years, including four male (66.67%) and two female (33.33%) patients. The mean age was 34.00 ± 16.73 years for male patients and 33.50 ± 9.19 years for female patients; the male-to-female ratio was 2:1.

**Table 2:** General data and clinical manifestations of six patients with ITB misdiagnosed as CD.

Variables	Number (*n*)	Percentage (%)
Gender		
Male	4	66.67
Female	2	33.33
Mean age	34.00 ± 16.73 years	
Symptoms		
Abdominal pain	5	83.33
Diarrhea	2	33.33
Fever	1	16.67
Fatigue	1	16.67
Emaciation	1	16.67
Vomiting	1	16.67
Chest tightness	1	16.67
Laboratory findings		
Elevated Leukocyte	1	16.67
Amenia	1	16.67
Hypoalbuminemia	1	16.67
Elevated CRP	2	33.33
Elevated ESR	2	33.33
PPD positive	3	50
T-SPOT.TB test positive	6	100
Imaging item		
Chest x-ray	1	16.67
Chest CT	5	83.33
Abdominal CT	2	33.33
Total gastrointestinal CT angiography	4	66.67
Colonoscopy result		
Segmental changes	5	83.33
Ulcerative colitis like changes	1	16.67
Pathological findings		
Granulomatous inflammation	5	83.33
Chronic inflammation	1	16.67

ITB, intestinal tuberculosis; CRP, C-reactive protein; ESR, erythrocyte sedimentation rate; PPD, purified protein-derived tuberculin, T-SPOT.TB test, tuberculosis infection based on the number of spot-forming T cells.

The main clinical manifestation was abdominal pain, followed by diarrhea, fever, fatigue, emaciation, vomiting, and chest tightness. Abdominal pain mainly consisted of lower abdominal or dull periumbilical pain. Diarrhea consisted of unformed loose stools occurring 2–5 times/day, without pus or blood in the stool (Table 2).

As shown in Table 2, the leukocyte count was elevated in one patient (16.67%), with a mean count of 7.70 ± 5.36 × 10^9^/L. Anemia was present in one patient (16.67%) and was moderate; the mean hemoglobin level was 124.83 ± 24.07 g/L. Platelet counts were normal, with a mean of 230.00 ± 22.63 × 10^9^/L. Hypoalbuminemia was present in one patient (16.67%), with a mean albumin level of 37.50 ± 7.62 g/L. Fecal occult blood testing was negative. CRP and ESR were each elevated in two patients (33.33%), and ESR was markedly elevated (>50 mm/h) in one patient. One patient had tuberculous pleurisy and pleural effusion. All six patients underwent PPD and T-SPOT.TB testing; the positive rates were 50% and 100%, respectively.

All six patients underwent chest imaging, including chest x-ray in one and chest CT in five. Active TB was detected in three patients and old TB in three. All six patients underwent abdominal imaging, including abdominal CT in two and total gastrointestinal CT angiography in four. In the four patients who underwent total gastrointestinal CT angiography, imaging showed segmental skip areas of wall thickening, suggesting CD.

**Figure 1:**
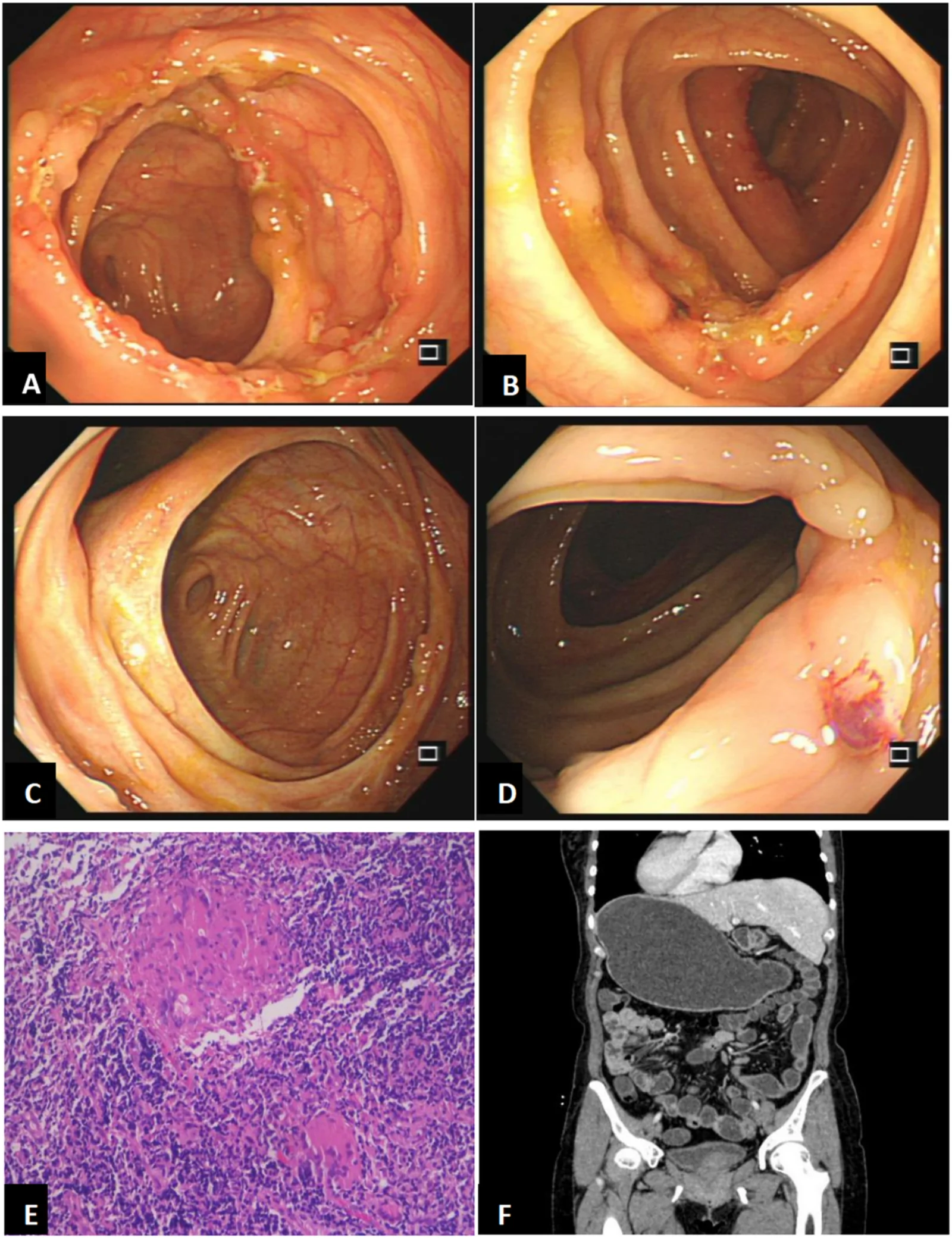
Endoscopic images before and after treatment of one ITB patients misdiagnosed with CD (case 1). **(A,B)** Colonoscopy showed segmental lesions in ileocecal part and ascending colon. The lesions are granuloid eminence, and the formation of fissure ulcers can be seen; **(C,D)** the ileocecal and ascending colon lesions were significantly improved after anti-tuberculosis treatment; **(E)** pathological findings showed granulomatous inflammation and multinuclear giant cell reaction; **(F)** a total gastrointestinal CT angiography showed that the intestinal wall was leaping and segmental thickening and strengthening, and the small intestinal mesenteric vascular hyperplasia showed “tooth dressing” change.

**Figure 2:**
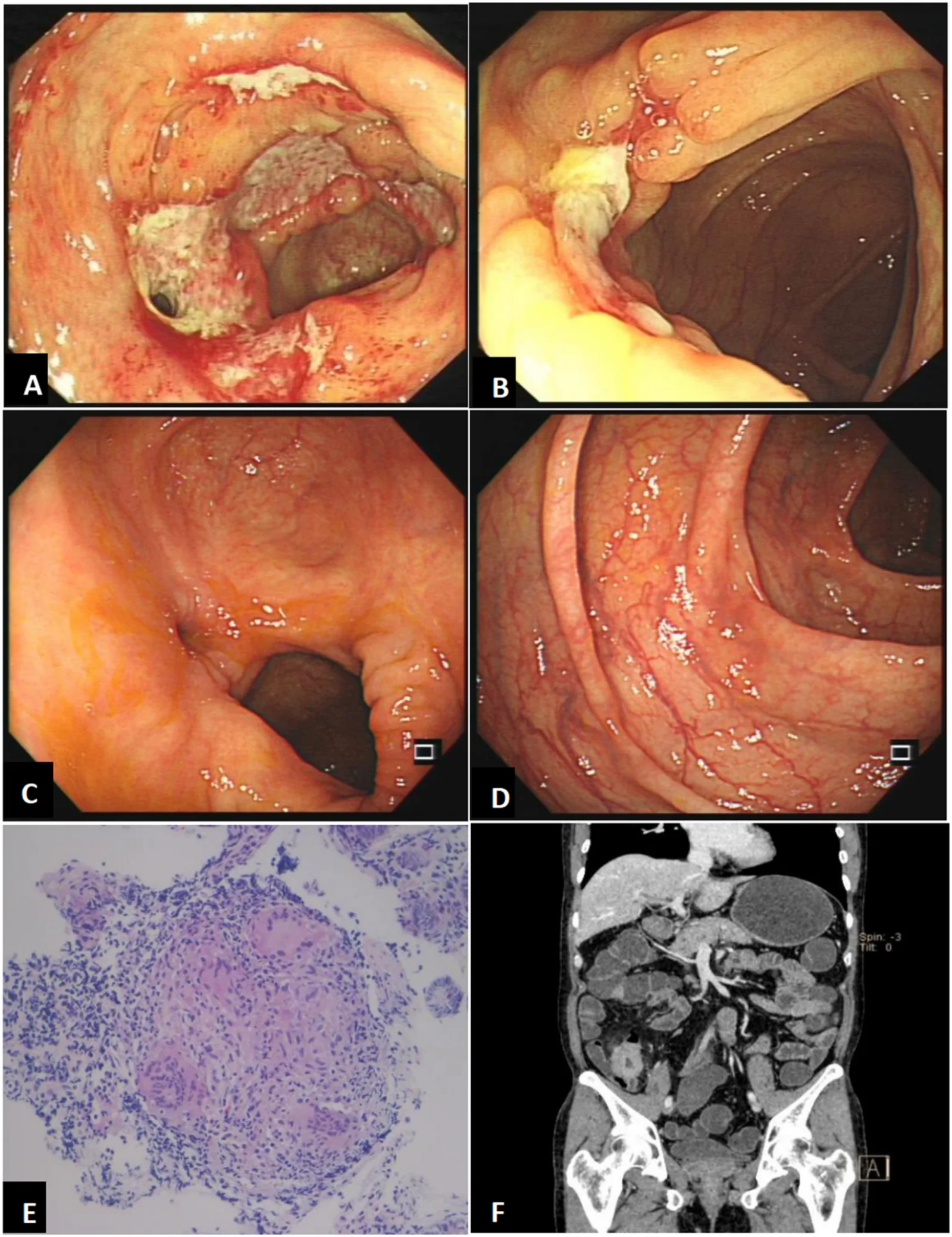
Endoscopic images before and after treatment of one ITB patient misdiagnosed with CD (case 2). **(A,B)** Colonoscopy showed that the ileocecal valve was deformed with stenosis, surface covered with ulcers, and annular or semi-annular ulcers were discontinuous from the transverse colon to the ileocecal part; **(C,D)** the ulcers from transverse colon to ileocecal valve were significantly improved after anti-tuberculosis treatment; **(E)** pathology showed non-necrotizing granulomatous inflammation with multinuclear giant cell reaction; **(F)** a total gastrointestinal CT angiography showed that the intestinal wall was leaping and segmental thickening and strengthening.

**Figure 3:**
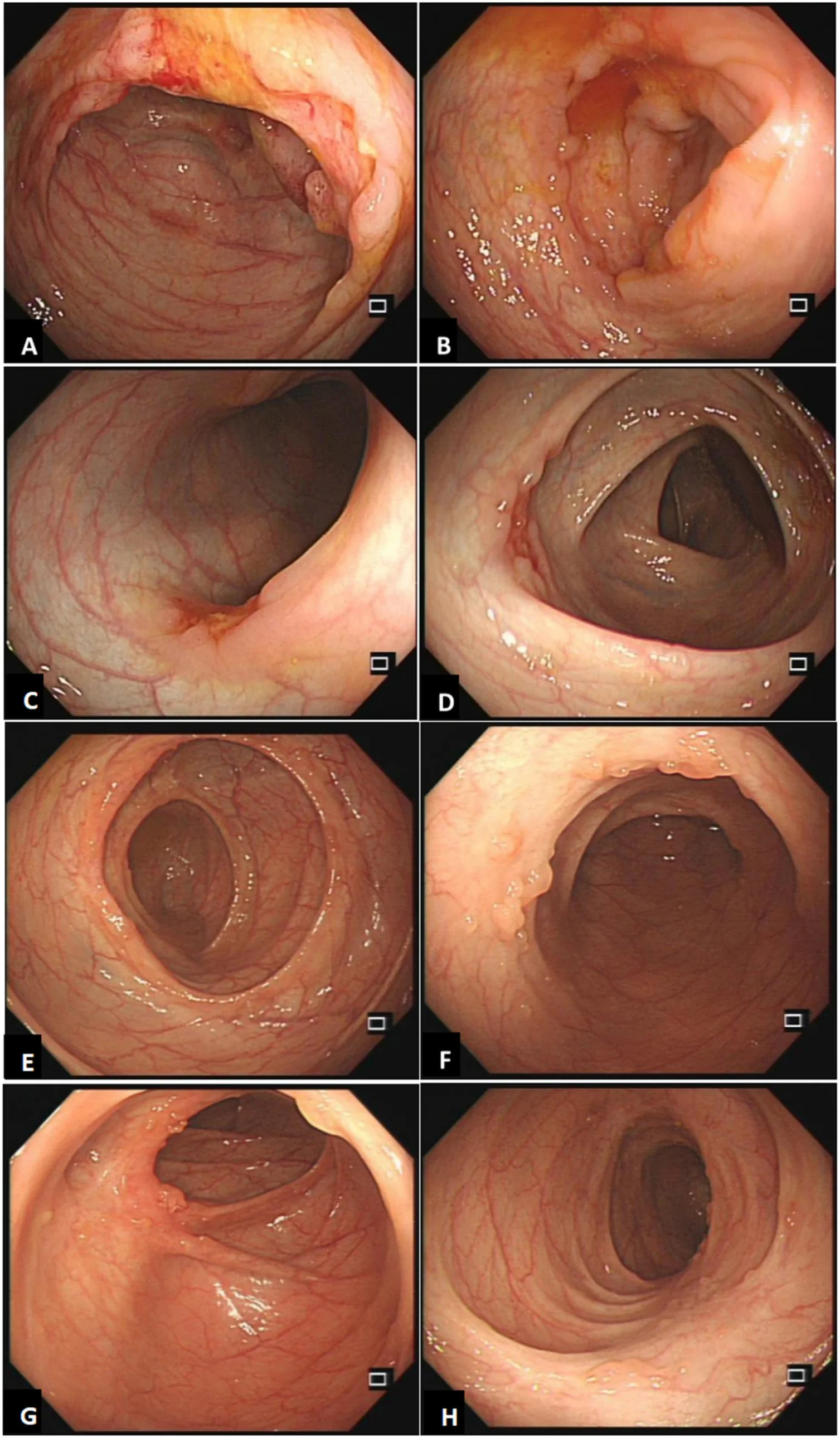
Endoscopic image of segmental changes in ITB patients misdiagnosed with CD. **(A–D)** colonoscopy revealed multiple irregular ulcers from sigmoid to ileocecal region; **(E–H)** colonoscopy showed multiple inflammatory pseudopolypoid ridges from the descending colon to the ileocecal part.

All six patients underwent colonoscopy. The endoscopic lesions primarily showed segmental changes in the colon. Figures 1–3 show endoscopic images of segmental intestinal changes in patients with ITB, and Figures 1, 2 show images obtained before and after treatment in patients with ITB initially misdiagnosed as CD. Histopathological examination showed granulomatous inflammation in five patients and mucositis without granulomas in one (Table 2).

Based on the total gastrointestinal CT imaging, colonoscopic, and pathological findings, IBD was strongly suspected. However, the laboratory and chest imaging findings did not allow TB to be excluded; therefore, all six patients received diagnostic antituberculosis therapy. Symptoms such as abdominal pain and diarrhea were significantly relieved after 6 weeks of treatment. After 6 months of antituberculosis therapy, endoscopy showed significant improvement in intestinal granulomatous hyperplasia and annular ulcers (Figures 1, 2).

## Discussion

ITB tends to occur in younger adults, most of whom are between 20 and 50 years old (20). In this study, 58.7% (27/46) of patients were between 20 and 50 years old, consistent with previous reports. The male-to-female ratio was approximately 1.6:1, indicating a predominance of male patients.

Abdominal pain, alternating diarrhea and constipation, and abdominal masses are generally considered the main clinical symptoms of ITB. In this study, the clinical manifestations of ITB were abdominal pain, abdominal distension, and diarrhea. Alternating diarrhea and constipation was once considered a clinical feature of ITB, but this manifestation is also common in other intestinal diseases. A small number of patients with ITB initially present with acute intestinal obstruction or perforation, which may be overlooked. In this study, seven patients (15.22%) received emergency treatment for acute abdominal symptoms, and 21 patients underwent surgery because of suspected intestinal tumors (three cases) or intestinal obstruction (18 cases). Thus, clinicians should be alert to atypical clinical manifestations of ITB and strive for early diagnosis and treatment to reduce the occurrence of complications.

A small proportion of ITB cases are primary, whereas most are secondary. It has been reported that 48%–69% of ITB cases are associated with pulmonary TB (21), compared with 34.78% in this study. Among the 46 patients, 20 had coexisting extraintestinal TB, of whom 16 had pulmonary TB, indicating that pulmonary TB was common among patients with extraintestinal TB. Chest x-ray and CT can detect pulmonary TB, providing important evidence for the diagnosis of ITB.

Laboratory and imaging examinations play a role in the diagnosis of ITB. In our study, the positive rates of the T-SPOT.TB test, PPD test, and chest imaging were 81.25% (26/32), 40.91% (9/22), and 39.13% (18/46), respectively. Abdominal imaging is of great value in the diagnosis of intestinal obstruction and perforation but has limited value in the diagnosis of ITB. The false-negative rate of the PPD test in this group was as high as 59.09%. The PPD test can be negative in the early stage of TB or when immunity is impaired; therefore, a negative PPD test cannot completely exclude the possibility of ITB (22). Shi et al. (23) reported that the positive rates of the T-SPOT.TB and PPD tests were 76.9% and 50.0%, respectively. Another study (24) showed that the sensitivity and specificity of the T-SPOT.TB test for ITB were 85.7% (12/14) and 93.3% (14/15), respectively. In terms of agreement, among the eight patients who tested positive for PPD, four also tested positive for T-SPOT.TB, indicating 50% agreement between the two diagnostic methods. Ferrara et al. (25) conducted a prospective study and reported an agreement rate of 75.9% between the T-SPOT.TB and PPD tests. The agreement rate in this study was much lower than that previously reported, which may be due to bias associated with the small sample size. In the subgroup of six patients with ITB misdiagnosed as CD, all six underwent PPD and T-SPOT.TB testing, and the positive rates were 50% and 100%, respectively, indicating 50% agreement between the two diagnostic methods. In general, the sensitivity and specificity of the T-SPOT.TB test are higher than those of the PPD test, which may improve the detection of TB infection, particularly for the rapid diagnosis and differential diagnosis of ITB.

Colonoscopic lesions in patients with ITB mainly involve the ileocecal region, ascending colon, and terminal ileum. In our study, the lesions mainly involved the ileocecal region (52.17%), terminal ileum (13.04%), and ascending colon (10.87%). Consistent with previous reports (26–28), typical ulcers and typical hypertrophic lesions were observed in 53.85% (7/13) and 62.5% (10/16) of cases, respectively, whereas the remaining 12 patients who underwent colonoscopy showed nonspecific ulcers that could easily be confused with IBD. However, chest CT in these 12 patients showed pulmonary TB. Therefore, patients with multiple colonic ulcers should undergo chest CT to assess for the possibility of ITB. If ITB is misdiagnosed as IBD, the use of immunosuppressants and glucocorticoids can lead to rapid dissemination of TB, resulting in hematogenous disseminated TB, nervous system TB, and other conditions associated with a poor prognosis. Therefore, some researchers believe that when patients have evidence supporting a diagnosis of TB, diagnostic antituberculosis therapy should be considered first so that the risk associated with misdiagnosis can be controlled and patients can derive greater benefit (29).

Pathological findings are considered the gold standard for the diagnosis of ITB; however, in this study, the positive rate of histopathological examination of colonoscopic biopsy specimens was low, with only 18 endoscopic biopsy specimens showing caseating granulomas and confirming ITB. The pathological findings in our study mainly consisted of granulomatous inflammation. Most researchers believe that the pathological changes of ITB occur mainly in the submucosa and that the depth of tissue obtained by endoscopic biopsy is insufficient; therefore, biopsy results mainly show nonspecific inflammatory changes (30, 31).

In the subgroup of six patients with ITB misdiagnosed as CD, all six mainly presented with segmental changes in the colon, and total gastrointestinal CT angiography in four patients showed segmental skip areas of wall thickening, suggesting the possibility of CD. Pathological examination showed granulomatous inflammation in five patients and mucositis without granulomas in one; none showed the characteristic finding of caseating granulomas. However, chest imaging was performed in all six patients and showed active TB in three and old TB in three. Based on comprehensive consideration of the laboratory, imaging, endoscopic, and pathological findings, ITB could not be ruled out, and all six patients received diagnostic antituberculosis therapy. After treatment, the patients' symptoms were significantly relieved, and endoscopic findings showed significant improvement in intestinal ulcers and inflammatory pseudopolyp hyperplasia.

The key to reducing misdiagnosis is to improve the recognition of ITB. The common features of the six misdiagnosed cases in this study suggest that patients with ITB may be particularly likely to be misdiagnosed as having CD when they have the following three characteristics: endoscopy shows multiple segmental lesions in the colon extending beyond the ileocecal region, histology lacks typical caseating granulomas and shows only nonspecific granulomatous inflammation or simple mucosal inflammation, and microbiological examination is negative at initial diagnosis and systemic symptoms of TB are not prominent. However, the effectiveness of diagnostic antituberculosis treatment is the most persuasive clinical evidence for distinguishing ITB from CD. All six patients in this group showed significant and persistent clinical and endoscopic remission after antituberculosis treatment. This response is usually not observed in patients with CD. Therefore, when clinical suspicion of ITB is high but microbiological evidence is lacking, prompt initiation of diagnostic antituberculosis treatment and close follow-up after other diseases have been fully excluded are key strategies for reducing misdiagnosis.

As shown in Table 3, based on the lessons learned from this group of cases, we suggest that for patients from areas with a high incidence of TB who have segmental lesions in the colon on endoscopy with prominent involvement of the ileocecal region, ITB should still be regarded as one of the primary differential diagnoses even if histology does not show caseous necrosis. Microbiological and molecular tests (such as tissue PCR or Xpert MTB/RIF) should be performed early, and diagnostic antituberculosis treatment should be given when necessary.

**Table 3:** High-risk clinical features associated with initially misdiagnosis of ITB as CD (*n* = 6).

Feature dimension	Misdiagnosed subgroup characteristics	Proportion (%)	Clinical implication
Endoscopic distribution	Segmental or skip lesions beyond the ileocecal region	100 (6/6)	Atypical distribution mimicking CD; deviates from the classic ileocecal-predominant pattern of ITB
Histopathology	Absence of caseating granuloma	100 (6/6)	Loss of the “gold standard” pathological clue, complicating definitive diagnosis
Microbiological tests	Negative acid-fast staining (sputum) and negative fecal occult blood	100 (6/6)	Lack of etiological evidence at initial presentation
Radiological findings	Abdominal imaging initially interpreted as CD-like changes	66.7 (4/6)	Radiological overlap between ITB and CD
Response to anti-tuberculosis therapy	Significant clinical and endoscopic improvement after anti-tuberculosis therapy	100 (6/6)	The most compelling functional evidence supporting ITB diagnosis and the key differentiator from CD

ITB, intestinal tuberculosis; CD, Crohn's disease.

Several limitations of this study should be acknowledged. First, this was a single-center, retrospective descriptive analysis involving only 46 patients with ITB, including six cases in the misdiagnosis subgroup. The sample size was limited. Second, no CD control group was established, making it impossible to conduct statistical comparisons between groups. Further validation is needed in multicenter, prospective studies with larger sample sizes.

## Conclusions

ITB can easily be confused with CD. Diagnosis requires a comprehensive assessment of clinical manifestations and endoscopic, imaging, pathological, and microbiological findings. The effectiveness of diagnostic antituberculosis treatment is an important basis for differentiation. In this group of six misdiagnosed cases, all patients improved significantly after antituberculosis treatment. This finding suggests that in atypical cases, diagnostic treatment should be initiated as early as possible and close follow-up should be performed.

## Data Availability

The original contributions presented in the study are included in the article/Supplementary Material, further inquiries can be directed to the corresponding author.
